# In Vitro Evaluation of a Vibrating-Mesh Nebulizer Repeatedly Use over 28 Days

**DOI:** 10.3390/pharmaceutics12100971

**Published:** 2020-10-15

**Authors:** Hui-Ling Lin, Chi-Shuo Chen, James B. Fink, Guo-Hao Lee, Chun-Wei Huang, Jui-Chi Chen, Zi Yi Chiang

**Affiliations:** 1 Department of Respiratory Therapy, Collage of Medicine, Chang Gung University, Taoyuan 33301, Taiwan; 0916brad@gmail.com (G.-H.L.); eric8501better@gmail.com (C.-W.H.); rachelchen4895@gmail.com (J.-C.C.); sara841009@gmail.com (Z.Y.C.); 2 Department of Respiratory Therapy, Chang Gung Memorial Hospital, Chiayi 61301, Taiwan; 3 Department of Respiratory Care, Chang Gung University of Science and Technology, Chiayi 61301, Taiwan; 4 Department of Biomedical Engineering and Environmental Sciences, National Tsing Hua University, Hsinchu City 30013, Taiwan; chen.cs@mx.nthu.edu.tw; 5 Aerogen Pharma Corp., San Mateo, CA 94043, USA; fink.jim@gmail.com; 6 Department of Respiratory Care, University of Texas, Round Rock, TX 78665, USA

**Keywords:** reused vibrating-mesh nebulizer, inhaled dose, particle size distribution, residual volume

## Abstract

This in vitro study evaluates the performance of a disposable vibrating-mesh nebulizer when used for 28 days. A lung model was used to simulate the breathing pattern of an adult with chronic obstructive pulmonary disease. The vibrating-mesh nebulizer was used for three treatments/day over 28 days without cleaning after each test. Results showed that the inhaled drug dose was similar during four weeks of use (*p* = 0.157), with 16.73 ± 4.46% at baseline and 15.29 ± 2.45%, 16.21 ± 2.21%, 17.56 ± 1.98%, and 17.13 ± 1.81%, after the first, second, third, and fourth weeks, respectively. The particle size distribution, residual drug volume, and nebulization time remained similar across four weeks of use (*p* = 0.110, *p* = 0.763, and *p* = 0.573, respectively). Mesh was inspected using optical microscopy and showed that approximately 50% of mesh pores were obscured after 84 runs, and light penetration through the aperture plate was significantly reduced after the 21st use (*p* < 0.001) with no correlation to nebulizer performance. We conclude that the vibrating-mesh nebulizer delivered doses of salbutamol solution effectively over four weeks without cleaning after each use even though the patency and clarity of the aperture plate were reduced by the first week of use.

## 1. Introduction

The administration of aerosolized drugs using a vibrating-mesh nebulizer has gained popularity because of its high delivery efficiency, low residual dose, and lack of requirement for external gas power [1,2]. A vibrating-mesh nebulizer utilizes a vibrating piezo-element and moves the drug solution through a mesh aperture plate to generate aerosols [3,4]. The advantages of vibrating-mesh nebulizers over the jet and other ultrasonic nebulizers include less noise, effective concentration, and minimal temperature changes that make it suitable for temperature-sensitive drugs. Vibrating-mesh nebulizers are predominantly used in clinical trials involving mechanical ventilation and newly developed medications [2,3,5,6,7,8].

The performance of a vibrating-mesh nebulizer can be affected by numerous factors, including viscosity and electrolyte conductivity of the solution, and clogging of the mesh plate by drug crystallization [9,10,11]. Beck-Broichsitter et al., evaluated vibrating-mesh nebulizer performance using solutions of different viscosities and reported that particle size measured in volume median diameter decreased as the solution viscosity and nebulizer output rate increased [12]. They further illustrated that the aerosol particle size decreased with increasing sodium chloride concentration that increased ion conductivity [10,11]. Ghazanfari et al., compared the performance of vibrating-mesh nebulizer using solutions of different viscosities and surface tensions and showed that low viscosity and reduced surface tension shortened nebulization time and increased the output rate [13]. Their results showed no association between fluid properties and nebulizer output, while the aerosol particle size increased as viscosity decreased. Higher viscosity reduced aerosol generation by vibrating-mesh nebulizers. The electrolyte concentration influences solution conductivity and consequently impacts the output of the vibrating-mesh nebulizer. Electrolyte conductivity of formulations has a greater impact on the performance of vibrating-mesh nebulizers than jet nebulizers [14].

Clinicians are concerned that a vibrating-mesh nebulizer may malfunction owing to clogging or occlusion of the mesh plate. Previous research has suggested that suspensions or viscous drugs could clog the pores of a mesh nebulizer without making a noticeable difference to the nebulizer output [9]. Rottier et al., evaluated vibrating-mesh and jet nebulizers for the delivery of inhaled tobramycin in ten patients with cystic-fibrosis over six months [9]. Particle sizes (measured using eFlow, Pari Ltd., Starnberg, Germany) were very similar; however, aerosol generation was inconsistent owing to clogging by drug. There are several papers by investigators, as well as the manufacturer demonstrating device performance in a number of clinical applications with a wide range of formulations [3,5,6,7,8]. The intellectual property portfolio is largely focused on physical attributes of the technology, such as the number, shape and configuration of apertures, and the composition of both the mesh, the key components of the aerosol generator, as well as the design and function of the medical reservoir. The current gap in the literature remains the implications of extended multiple-dose use as described in the current device label. Gowda et al., evaluated the reliability of a vibrating-mesh nebulizer (Aerogen Solo, Aerogen Ltd. Galway, Ireland) by nebulizing 3 mL of normal saline with 20 nebulizers with each of two controllers, reporting that random interruption of aerosol generation resulted in a wide range of residual volume [15]. Evaluation of reliability and performance of the Aerogen Solo repeatedly used with consecutive treatments over extended periods of time has not been reported.

According to the manufacturer, the Aerogen Solo single-patient, a multiple-dose disposable vibrating-mesh nebulizer can be used for up to 28 days without cleaning between treatments. A consistent performance of the nebulizer is crucial for patients receiving aerosol therapy. Therefore, the aim of this in vitro study was to evaluate the performance of this vibrating-mesh nebulizer when repeatedly used over time.

## 2. Materials and Methods

### 2.1. Lung Model

**Figure 1:**
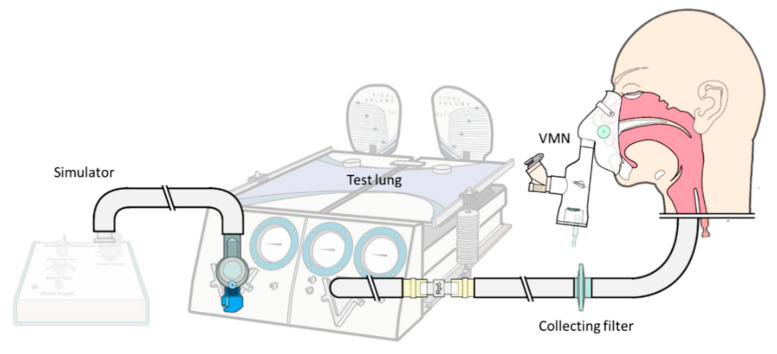
Apparatus of the experimental setup. A simulation module generated negative spontaneous breathing, and a collecting filter distal to the trachea of the mannequin collected aerosolized drug inhaled.

A dual-compartment passive test lung (Michigan Instruments Inc., Grand Rapids, MI, USA) consisting of two compartments connected with a rigid bar and a breath-simulation module, which inflates one compartment which lifts the other compartment, simulating a spontaneous breathing pattern (Figure 1). The model was set to simulate parameters of an adult patient with a chronic obstructive pulmonary disease, as follows: Tidal volume of 500 mL, respiratory rate 15 of breaths/min, inspiratory time of 1 s, lung compliance of 0.06 L/cm H_2_O, and airway resistance of 20 cm H_2_O/L/sec. A collecting filter was placed distal to the trachea of a teaching mannequin to capture aerosols, referred to as the inhaled dose.

### 2.2. Experiment Protocol

A vibrating-mesh nebulizer (Aerogen Solo, Aerogen Ltd., Dangan, Galway, Ireland) was selected for our experiments because of its label claim for extended use, ready availability, and use in hospital settings. The nebulizer was connected to a valve mask, and valve reservoir (Aerogen Ultra^TM^, Aerogen Ltd., Dangan, Galway, Ireland) placed on the face of the adult teaching mannequin; 2 L/min of oxygen was delivered to the oxygen port of the reservoir [15]. According to the user instructions, the nebulizer can be used to administer intermittent treatments for a maximum of 28 days. Therefore, we designed the experiment to administer aerosol treatments three times a day at 6-h intervals during the day for 28 days, representing a total of 84 treatments. Five new nebulizers operated for 28-days administration were used in a random order to administer a commercially available unit dose of salbutamol (5.0 mg/2.5, containing salbutamol, sodium chloride, dilute sulfuric acid, and water; mL, GlaxoSmithKline Inc., Victoria, Australia) placed in the nebulizer without further dilution. The nebulizers air-dried without washing or rinsing after each experiment. Each nebulizer was operated continuously with an electronic controller (Aerogen ProX, Aerogen Ltd., Dangan, Galway, Ireland), which was turned off when no emitted aerosol was seen.

Nebulizer performance consistency was determined by measuring the inhaled drug dose, aerosol particle size distribution, nebulization time, and residual volume. The inhaled drug dose distal to the bronchi of the model was collected on a bacterial filter. The nebulizer was weighed before treatment, after being filled with salbutamol, and after each treatment to determine residual volume, which was converted based on the density of salbutamol (1.001 g/mL). Additionally, the nebulization time was recorded.

The mass median aerodynamic (MMAD) was measured using an Anderson Cascade Impactor (ThermoFisher Scientific Inc., Waltham, MA, USA) during the 1st, 21st, 42nd, 63rd, and 84th run of each nebulizer, representing the performance after each week of use. The MMAD is defined as the diameter at which 50% of the particles by mass are larger and 50% are smaller, whereas the FPF corresponds to the fraction of drugs carried in particles with a diameter of <4.7 µm. The MMAD and geometric standard deviation (GSD) were calculated using the Copley Inhaler Testing Data Analysis Software (Copley Scientific Ltd., Nottingham, UK).

### 2.3. Drug Assay

The aerosolized drug deposited on the collecting filters distal to the trachea was eluted with 10 mL of distilled water with gentle agitation for 2 min. The collection plates of the Anderson Cascade Impactor were disassembled and eluted with 10 mL of distilled water. The absorbance of each drug sample was measured using an ultraviolet spectrophotometer (ThermoFisher Scientific Inc., Waltham, MA, USA) at a wavelength of 276 nm. There was a linear relationship between the absorption and concentration of salbutamol between 2.0 and 250 µg/mL with a slope of 0.0061 (*R*^2^ = 0.9999). Salbutamol drug mass was calculated from the absorption-concentration standard curve.

### 2.4. Aperture Plate Imaging 

To determine the effect of extended use on the nebulizer aperture plate, separate experiments were carried out to administer aerosol treatments three times a day at 6-h intervals during the day for 7 days and 28 days, representing one week and four weeks of use. A nebulizer pre-use, and nebulizers with 21 and 84 runs were disassembled, and the aperture plates were imaged using an inverted light microscope (Eclipse-Ti, Nikon Instruments Inc., New York, NY, USA). Image analysis was performed using ImageJ software. The patency of the aperture plates was determined by the penetration of the hole size and number of holes.

### 2.5. Statistical Analysis

The amount of drug deposited on the filter was expressed as the total fraction of the charged dose placed in each nebulizer. Descriptive statistics, including the mean and standard deviations, were calculated. Data were analyzed using Statistical Package for the Social Sciences version 24.0 (IBM Inc., New York, NY, USA). The performance of the nebulizer over time was assessed using repeated-measures analysis of variance of inhaled drug dose, particle size, nebulization time, and residual volume. Changes in light penetration among the three conditions were analyzed using analysis of variance. *p* values < 0.05 were considered to be statistically significant.

## 3. Results

### 3.1. Inhaled Drug Dose

**Figure 2:**
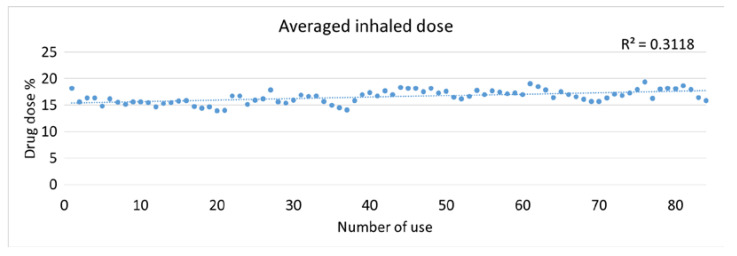
Averaged inhaled drug dose (%) over time (*n* = 5).

**Figure 3:**
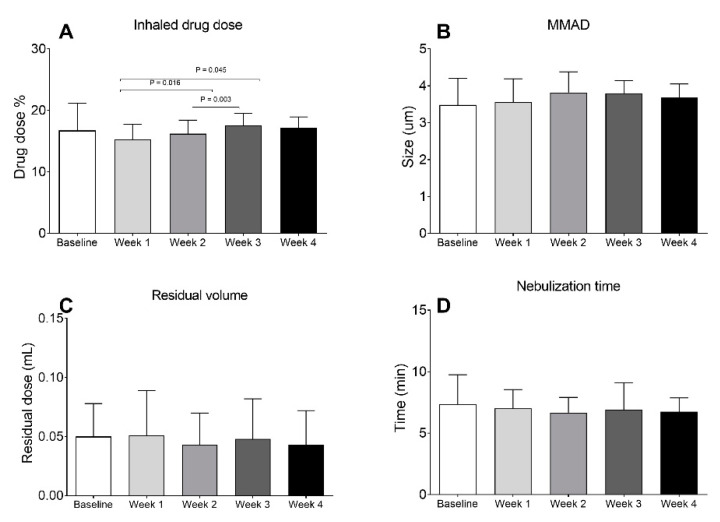
Comparison of nebulizer performance across four weeks of use: (**A**) Inhaled drug dose; (**B**) median mass aerodynamics (MMAD); (**C**) residual volume; and (**D**) nebulization time.

The average inhaled drug doses of 5 nebulizers were between 13.9% and 19.3% and showed a low correlation with the number of uses (Figure 2). Results of inhaled drug dose were grouped into five sections: The first test at baseline, the first week of use (2nd–21st runs), second week (22nd–42nd runs), third week (43rd–63rd runs), and fourth week (64th–84th runs). Figure 3A shows the comparison of inhaled drug dose from baseline to four weeks of use. The average inhaled drug doses were 16.73 ± 4.46% at baseline, 15.29 ± 2.45% at week 1, 16.21 ± 2.21% at week 2, 17.56 ± 1.98% at week 3, and 17.13 ± 1.81% at week 4 were similar with no observed trend (*p* = 0.157). Compared to week 1, the inhaled drug dose was significantly increased at week 3 (*p* = 0.016) and week 4 (*p* = 0.045).

### 3.2. Nebulizer Performance

Figure 3B shows that the MMAD (mean ± SD) ranged from 3.56–3.68 μm without significant difference across four weeks of use (*p* = 0.110). The geometric standard deviations (GSD) were 2.0 ± 0.1 at baseline, 2.04 ± 0.09 at the first week, 2.02 ± 0.08 at the second week, 2.02 ± 0.04 at the third week, and 2.04 ± 0.05 at the fourth week (*p* = 0.626). The fine particle faction (<4.7 μm) was 65.6 ± 5.4% at baseline, 64.7 ± 4.7% at the first week, 64.7 ± 4.5% at the second week, 58.4 ± 2.4% at the third week, and 60.5 ± 1.9% at the fourth week (*p* = 0.712). The residual volumes measured gravimetrically were similar across results obtained from 0.05 ± 0.02 µL at baseline, 0.05 ± 0.03 µL at week 1, 0.04 ± 0.03 µL at week 2, 0.05 ± 0.03 µL at week 3, and 0.04 ± 0.03 µL at week 4 (Figure 3C), without statistical difference (*p* = 0.763). Lastly, the nebulization times were 7.37 ± 2.38 min at baseline, 7.01 ± 1.53 min at the first week, 6.74 ± 1.24 at the second week, 6.9 ± 1.21 min at the third week, and 6.74 ± 1.12 min at the fourth week (Figure 3D). The nebulization time tended to decrease with use, but no statistically significant difference, was found (*p* = 0.573).

### 3.3. Images of Aperture Plates 

**Figure 4:**
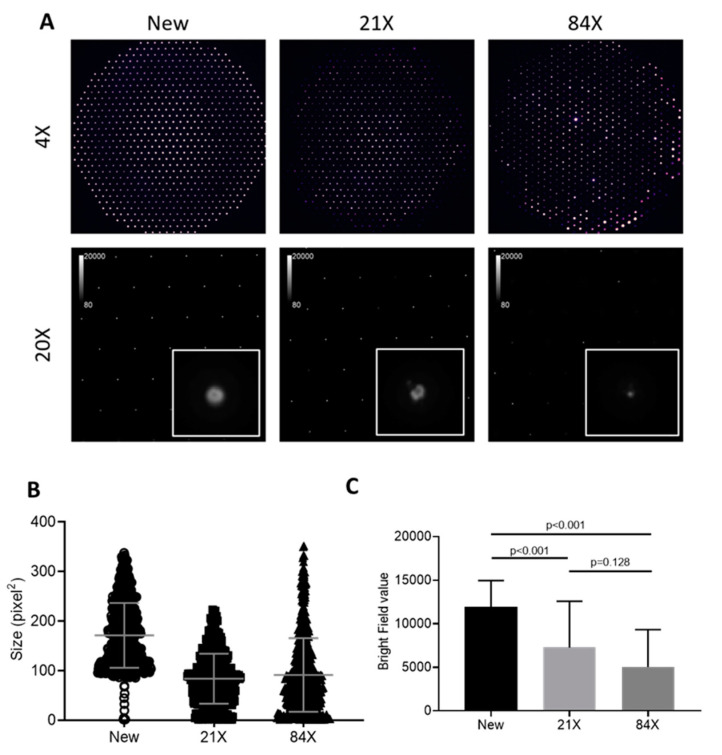
(**A**) Aperture plates with magnification 4× and 20× of three conditions, (**B**) total size of the opening area, and (**C**) intensity measurement of light penetrated through the mesh.

The images of aperture plates nebulizers unused and after 21 and 84 runs are shown in Figure 4A at 4× and 20× magnification. Figure 4B shows the size of the open area quantified in pixel square on the aperture plate. The medium size of the total holes was 161 pixel^2^ for the new aperture, 79 pixel^2^ for 21 runs, and 76 pixel^2^ for 84 runs (*p* < 0.001). We measured the total penetrated light intensity to represent mesh penetration, reflecting the number of holes and open area of holes. Figure 4C compares the bright field among the three apertures, documenting a 42% decrease for 21 runs and a 40% decrease for 84 runs compared to that of the new aperture (*p* < 0.001).

## 4. Discussion

Our study evaluated the performance of a repeatedly used vibrating-mesh nebulizer for 84 administrations of salbutamol over 28 days without rinsing or washing between treatments. Performance tests showed device reliability and performance consistency over 28 days of use regarding the inhaled dose, residual volume, particle size, and nebulization time. The images of aperture plates were partially occluded by the first week of use, but no further occlusion, was observed by the end of the 28-day use, with no direct correlation to reduced performance.

### 4.1. Nebulizer Performance

The effectiveness of a vibrating-mesh nebulizer has been extensively studied compared to other types of nebulizers. Only a few studies have assessed the reliability of vibrating-mesh nebulizers. Gowda et al., determined the reliability of a vibrating-mesh nebulizer (Aerogen solo™) using 3 mL normal saline by measuring the residual volume and nebulization time of 20 nebulizers each used with two controllers [15]. They reported residual volumes of 3.2 ± 1.5% for reliable nebulizers and 36 ± 21.3% for “failed” devices, with nebulization time ranging from 9.6–11.3 min. In contrast, with five nebulizers each used 89 times (5 for particle-size measurements and 84 for drug dose collection), we found a residual volume range of 0.04–0.05 µL (0.2% of charged volume), nebulization of 7.37 min regardless of the number of repeated uses, with no failures. Our output rate of 0.33–0.36 mL/min was similar to the 0.3–0.31 mL/min reported for reliable nebulizers in Gowda’s study, which are both consistent with the 0.3 mL/min label claim by the manufacturer.

Rottier et al., evaluated the performance of the eFlow vibrating mesh nebulizer device administering tobramycin for six months and reported that the average nebulization time was longer when the nebulizer was not cleaned after use [9]. The volume median diameter was similar between the new and used-cleaned nebulizers, but larger with the used-uncleaned nebulizer. The study reported inter-device variation hypothesized to be associated with clogging of the orifices of the drilled holes. The present study demonstrated consistent inhaled drug dose and particle size distribution delivered by the Aerogen Solo nebulizer. The eFlow device is designed for long-term use, and is recommended that the device is disassembled and cleaned immediately after each treatment. The cleaning process is user-dependent; thus, greater variations in nebulizer performance occur after long-term use. In contrast, the Aerogen Solo nebulizer is designed for single-patient use for up to 28 days when placed inline of a mechanical ventilator system or used on the Ultra spacer, with no cleaning being required. The user manual of the nebulizer indicates that MMAD is 3.4 µm with a GSD of 2.09. Our results showed that the MMAD at the baseline was 3.48 µm and the GSD of 2.02, similar to the manufacturer’s claim. There were no significant differences from baseline to the end of 4-week use (3.48 µm, 3.56 µm, 3.82 µm, 3.80 µm, 3.68 µm after the first, second, third, and fourth weeks, respectively).

### 4.2. Aperture Image

The Solo nebulizer aperture plate consists of 1000 funnel-shaped apertures. Microscopy images showed apparent occlusion or obscuration of apertures after 21 doses administered. From images with lower magnification, we observed heterogeneous obscuration patterns. Obscured apertures appeared aggregated, with a diameter of several hundred-micron areas—which suggests that clogging may alter local aerosol-flow distribution. At a higher magnification, more apertures showed irregular peripheries and appeared to be partially clogged or obscured, presumably with solute crystals, after extended use. We found that the obscuration level increased with usage time, but there was no obvious correlation between obscuration levels and inhaled doses, MMAD, and time of administration. This observation seemed consistent with previous reports that clogging did not significantly change the dose output of a mesh nebulizer [16]. Only a fraction of apertures actively pump drug-producing aerosol, suggesting that clogging might form with less active apertures. Two mechanisms may contribute to the dose output being consistent. First, the solute crystals deposited on the mesh obscured apertures may be re-dissolved with the introduction of the next dose. Second, the mesh vibration and pumping action not only facilitates aerosol generation, but may also physically aid clog removal from apertures. Additional experiments, including rinsing nebulizers prior to imaging, are needed to examine these possible mechanisms for obscuration.

### 4.3. Clinical Implication

As the vibrating-mesh nebulizer is considered relatively expensive compared to jet nebulizers, the ability to use for extended periods is critical for care providers to assess the cost/benefit for its use with their patients.

According to the manufacturer, the mesh nebulizer has approximately 1000 apertures. Not all apertures actively generate aerosol at the same time. It is unclear whether the perception of diminishing hole size is due to blockage of the apertures by crystals formed by drying out of residual albuterol and saline. This residual is possibly reconstituted with the addition of the next dose. With the low residual drug volume of the nebulizer, these reconstituted “crystals” would add a small amount of active drug to the subsequent dose. We calculated a maximum dose of 5.102 mg at dose 84. Additionally, while inhaled doses between each week appeared statistically significant, the differences would be too small to be of practical importance. The variation in MMAD, and inhaled dose would likely not impact clinical response.

Although the images of aperture plates appear to document partial occlusion after one week of use when not cleaned between treatments, the inhaled drug dose and nebulizer performance was similar across the four weeks of repeated use. This suggests that the staff, when administering aerosol treatment, can place the mesh nebulizer in the ventilator circuit without removing between doses to clean or add medication, avoiding “breaking the circuit”, which interrupts ventilation and can generate aerosols of contaminated condensate with explosive depressurization of the ventilator circuit, which may transmit infectious agents to care providers and others in the vicinity of the patient [17]. During the COVID-19 pandemic, or treatment of any patient infected with a pathogen transmitted by droplets or aerosol, the less manipulation and contact between the healthcare staff and patients, the lower the chance of the staff getting infected with the highly contagious pathogens.

### 4.4. Limitations

While our results demonstrated that the performance of the mesh nebulizer was consistent over 28 days of repeated use, we did not address potential contamination of the nebulizer over this period of time and use without cleaning. There are several implications for contamination with repeated nebulizer use [17]. Primary sources of contamination have been identified as are the patient and the health care provider Bioaerosols, and contaminated secretions from the patient can enter the medication reservoir. Health care provider use of the inadequate aseptic technique in handling, cleaning, and dispensing medication have been described. Standard jet and ultrasonic nebulizers have open medication reservoirs positioned below the level of the mouthpiece and circuit connected to the patient. In contrast, the mesh forms a barrier between the patient and the medication reservoir, which is of necessity positioned above the conduit or circuit connected to the patient airway, reducing the risk for patient-generated secretions or bioaerosols to contaminate the medication reservoir. Additionally, large residual drug volumes remaining in the jet and ultrasonic nebulizers offer a wet environment for pathogens to grow, resulting in CDC recommendations to wash, disinfect, sterilize, or air dry between treatment. The smaller residual drug volume (microliters vs. milliliters) with the mesh nebulizer allow for evaporation of liquid between doses—hence the formation of crystals that were potentially blocking apertures. This presents less opportunity for waterborne pathogen survival and proliferation.

Our experiments were performed using just one brand and model of vibrating-mesh nebulizer, and our findings should not be extended to other mesh nebulizers without additional testing. We chose this particular brand of vibrating-mesh nebulizer because it is widely used to treat acutely ill and intubated patients during mechanical ventilation. The inhaled drug dose is influenced by various factors during mechanical ventilation; therefore, further study on drug delivery in a ventilator system is warranted. The particle size testing was based on salbutamol solution, which is the most commonly administered drug used with nebulizers [13,18]. Further testing of a repeatedly used vibrating-mesh nebulizer with suspensions and viscous drug solutions, such as antibiotics, are warranted.

## 5. Conclusions

This study illustrates that the nebulization time, residual volume, and particle size of the vibrating-mesh nebulizer used without cleaning were consistent and reliable after repeated use over 28 days, despite the appearance of obscuration or partial clogging of apertures. Further studies on the repeated use of various liquid medications, such as antibiotics or budesonide suspension, are warranted.
